# Exploring the Molecular Mechanism underlying the Stable Purple-Red Leaf Phenotype in *Lagerstroemia indica* cv. Ebony Embers

**DOI:** 10.3390/ijms20225636

**Published:** 2019-11-11

**Authors:** Zhongquan Qiao, Sisi Liu, Huijie Zeng, Yongxin Li, Xiangying Wang, Yi Chen, Xiaoming Wang, Neng Cai

**Affiliations:** Hunan Academy of Forestry, 658 South Shaoshan Road, Changsha 410004, China; qiaozhongquan110@163.com (Z.Q.); liusisi274@126.com (S.L.); run507@163.com (H.Z.); yh5403@sohu.com (Y.L.); S13617494939@163.com (X.W.); lotofa@163.com (Y.C.)

**Keywords:** *Lagerstroemia indica*, gene expression, ornamental value, anthocyanins, leaf coloration, directional improvement

## Abstract

*Lagerstroemia indica* is an important ornamental tree worldwide. The development of cultivars with colorful leaves and increased ornamental value represents one of the current main research topics. We investigated the anthocyanin profiles in two contrasting cultivars for leaf color phenotypes and explored the underlying molecular basis. Both cultivars display purple-red young leaves (Stage 1), and when the leaves mature (Stage 2), they turn green in HD (Lagerstroemia Dynamite) but remain unchanged in ZD (Lagerstroemia Ebony Embers). Seven different anthocyanins were detected, and globally, the leaves of ZD contained higher levels of anthocyanins than those of HD at the two stages with the most pronounced difference observed at Stage 2. Transcriptome sequencing revealed that in contrast to HD, ZD tends to keep a higher activity level of key genes involved in the flavonoid–anthocyanin biosynthesis pathways throughout the leaf developmental stages in order to maintain the synthesis, accumulation, and modification of anthocyanins. By applying gene co-expression analysis, we detected 19 key MYB regulators were co-expressed with the flavonoid–anthocyanin biosynthetic genes and were found strongly down-regulated in HD. This study lays the foundation for the artificial manipulation of the anthocyanin biosynthesis in order to create new *L. indica* cultivars with colorful leaves and increased ornamental value.

## 1. Introduction

*Lagerstroemia indica* L. is a deciduous shrub and small tree of the genus Lagerstroemia with a great ornamental value thanks to its attractive blossom, long-lasting flowering period, and vase-shaped features [1]. It originated in China and has long been used in landscaping in major cities, including Anyang, Fuyang, and Jincheng [2]. *L. indica* cultivars have a wide range of flower colors (white, red, purple, and their combined variants), which contrast with a dark green foliage. However, the few existing cultivars with both colorful flowers and leaves have attracted a great interest and are the prime choice on the market [3]. Therefore, the development of new cultivars with colorful leaves and increased ornamental value has become one of the key research directions in breeding programs. In line with this, the United States Department of Agriculture has released the cultivar ‘Lagerstroemia Ebony Embers’, which has stable purple-red leaves throughout its leaf development [4]. So far, efforts to develop new *L. indica* cultivars have been mainly based on traditional breeding techniques [5,6,7,8]. Hence, it is still tedious to achieve the directional improvement of leaf color in *L. indica*. It is expected that modern molecular techniques will considerably facilitate and accelerate the improvement of leaf color in *L. indica* [9]. However, this requires a detailed understanding of the molecular mechanism of color formation in leaves of *L. indica*.

Color formation is one the most investigated and fascinating research questions in ornamental plants. Flavonoids, particularly anthocyanins, have been reported as the main coloring pigments in plants [10]. Anthocyanins provide a large spectrum of colors ranging from orange/red to violet/blue. Over the past decades, numerous works have clarified the biosynthetic pathway of anthocyanins, which is a very well-conserved network in plant species [11,12]. The key structural genes that catalyze the early and late steps of anthocyanin biosynthesis have been revealed and include phenylalanine ammonia-lyase (PAL), chalcone synthase (CHS), chalcone isomerase (CHI), flavonone 3-hydroxylase (F3H), flavonoid 3’-monooxygenase (F3′H), dihydroflavonol 4-reductase (DFR), anthocyanin synthase (ANS), and UDP-glucose-flavonoid 3-*O*-glucosyltrasnferase (UFGT) [13]. The specific variation in the expression levels of these structural genes through various and complex regulation mechanisms results in quantitative and qualitative variations of anthocyanins, underlying the difference of colorations observed between species, genotypes, organs, or even between various positions on the same plant tissue. Transcription factors (TF) such as MYB, basic helix loop-helix, and WD40 genes were reported to be the key modulators of the anthocyanin biosynthetic structural genes [14,15,16], but other regulators belonging to the TF families of WRKY and NAC have also been discovered [17,18,19]. Moreover, recent studies have demonstrated that genetic mutations and microRNAs represent other forms of regulation of the anthocyanin biosynthetic genes [20,21]. The species-specific peculiarity of anthocyanin regulation mechanisms justifies the numerous studies on color formation in plants.

The overall goal of this study is to clarify the molecular mechanism of color formation in leaves of *L. indica*. To achieve this objective, we explored the key anthocyanins conferring the purple-red color in leaves of ‘Lagerstroemia Ebony Embers’ compared to the cultivar ‘Lagerstroemia Dynamite’, which features green-colored mature leaves. In addition, we investigated the competition mechanism between different branches of anthocyanin biosynthesis and the TFs regulating the anthocyanin biosynthetic genes. The findings from this study will guide the artificial manipulation of the anthocyanin biosynthesis in order to create new cultivars with colorful leaves and increased ornamental value.

## 2. Results

### 2.1. Anthocyanin Analysis in the Leaves of the Two *Lagerstroemia indica* Cutlivars

**Figure 1:**
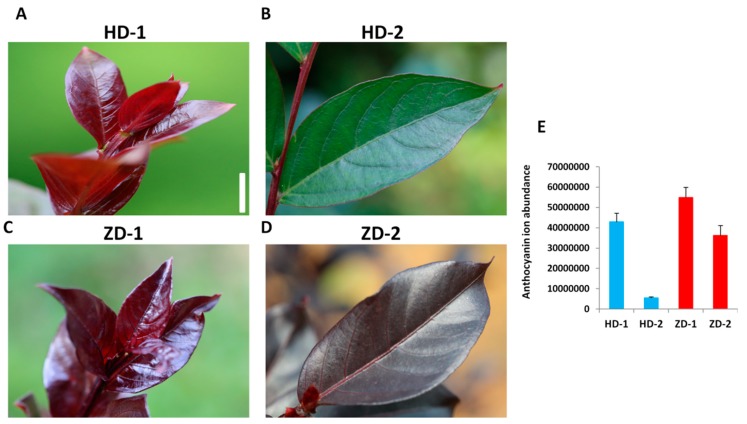
The phenotypes of young (**A**,**C**) and mature (**B**,**D**) leaves of HD and ZD. The bar represents 1.2 cm. (**E**) Total anthocyanin content measured in leaves of HD and ZD at young (Stage 1, S1) and mature (Stage 2, S2) stages. HD represents the cultivar Lagerstroemia Dynamite, while ZD represent the cultivar Lagerstroemia Ebony Embers. Data are from three replicate samples and represent the ion abundance of the anthocyanin compounds.

Two cultivars of *Lagerstroemia indica* with different leaf color phenotypes were studied. The two cultivars display purple-red young leaves (Stage 1), and when the leaves mature (Stage 2), they turn into green color in HD (Lagerstroemia Dynamite) but remain unchanged in ZD (Lagerstroemia Ebony Embers) (Figure 1A–D). Anthocyanins are known to be the major coloring pigments in plants [10]. We characterized the anthocyanin contents in the leaf samples of the two cultivars at the two stages of development. Seven anthocyanins, including peonidin O-hexoside, rosinidin O-hexoside, cyanidin O-syringic acid, cyanidin 3-O-glucoside (kuromanin), delphinidin 3-O-glucoside (mirtillin), cyanidin 3,5-O-diglucoside (cyanin), and cyanidin were detected (Table S1). Quantitative profiles showed that cyanidin was only detected in the leaves of HD, while the six other anthocyanins were present at different concentrations in the two cultivars. Globally, the leaves of ZD contained higher levels of anthocyanins than those of HD at the two stages with the most pronounced difference observed at Stage 2 (Figure 1E). In addition, the total anthocyanin content decreased from Stage 1 to Stage 2 in both cultivars, but the decrease was more conspicuous in HD (Figure 1E). This suggests that the leaf color change observed at Stage 2 in HD is associated with a significant decrease of total anthocyanins. Next, the concentrations of each metabolite were compared between the two cultivars (ZD-1_vs_HD-1 and ZD-2_vs_HD-2) and between the two developmental stages (HD-1_vs_HD-2 and ZD-1_vs_ZD-2) in order to identify the differentially accumulated metabolites (DAM) with the following parameters: variable importance in projection ≥1 and fold change ≥2 or fold change ≤0.5 [22]. In total, we found five, two, four, and seven DAM for HD-1_vs_HD-2, ZD-1_vs_ZD-2, ZD-1_vs_HD-1, and ZD-2_vs_HD-2, respectively (Table 1). This result further supports the premise that a strategy toward maintaining a higher content of all detected anthocyanins (except cyanidin) in ZD underpins the stable leaf coloration observed throughout the developmental stages.

### 2.2. De Novo Transcriptome Assembly and Gene Expression Profiles in the Two *L. indica* Cutlivars at Different Leaf Developmental Stages

**Figure 2:**
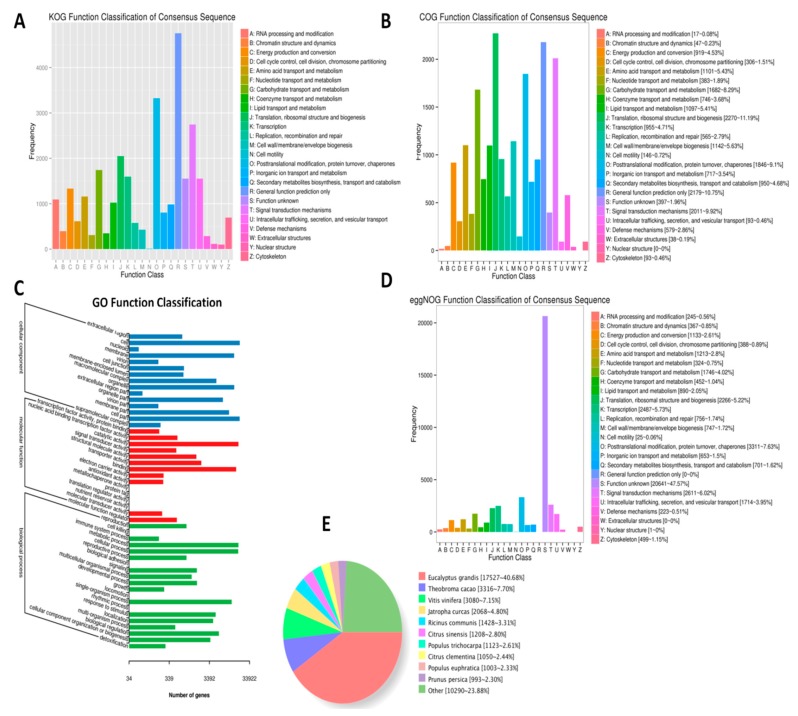
Functional annotation of the unigenes detected in *L. indica* based on orthologs from various databases. (**A**) KOG; (**B**) COG; (**C**) GO; (**D**) EggNOG; and (**E**) NR database homologous species distribution analysis.

In order to decode the genes involved in the differential leaf color phenotype in HD and ZD, we de novo sequenced and assembled the transcriptome from leaf samples of the two cultivars at the two stages and in triplicate. In total, 12 RNA-seq were generated, yielding a total of 283 millions reads and 84 Gb of clean data with 94% of bases scoring Q30 and above (Table 2). Using the Trinity software, 45,925 unigenes were assembled. To predict the functions of these genes, various databases were searched, including COG (18475), GO (33922), KEGG (17473), KOG (26429), Pfam (36768), Swiss-Prot (32110), eggNOG (42527), and NR (43088), resulting in a total of 43,208 functionally annotated genes (Figure 2A–D). NR database homologous species distribution analysis showed that *L. indica* (Lythraceae) shares 40% of its genes with *Eucalyptus grandis* (Myrtaceae), both species belonging to the same order: Myrtales (Figure 2E). We searched for genes encoding transcription factors (TF) and obtained a total of 2504 TFs classified into various families (Table S2).

**Figure 3:**
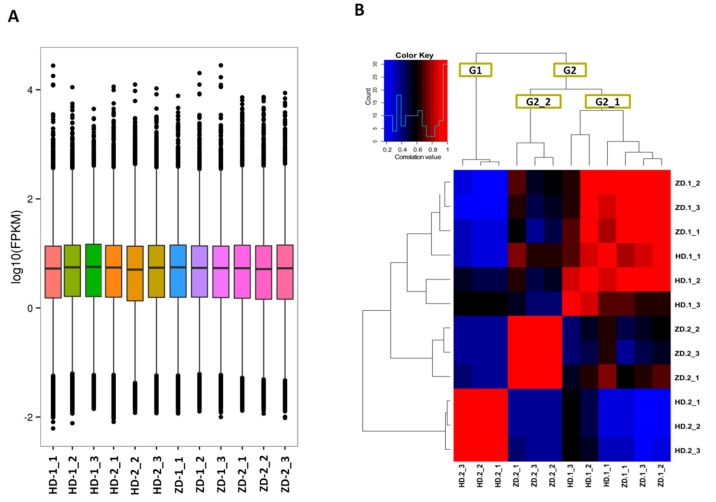
Overview of the transcriptome sequencing in *L. indica* leaves. (**A**) Gene expression profiles in the 12 libraries. HD represents the cultivar Lagerstroemia Dynamite, while ZD represents the cultivar Lagerstroemia Ebony Embers; (**B**) heatmap clustering showing correlation among samples based on global expression profiles. G1 and G2 represent the two major groups of samples, while G2_1 and G2_2 represent the subgroups of the group G2.

Gene expression levels were estimated with the fragments per kilobase of exon per million fragments mapped (FPKM) values ranging from 0.01 to 786,889 (Figure 3A). To assess the quality of the replicate samples, we performed hierarchical clustering analysis based on FPKM data. The result showed that all the biological replicates clustered together, suggesting a high reliability of our sequencing data (Figure 3B). Moreover, two main groups were displayed, including one group (G1) for the green-colored leaf samples (HD-2) and one group (G2) for the purple-red-colored samples (HD-1, ZD-1 and ZD-2). Also, G2 could be split into two subgroups, including G2_1 gathering samples from the young stage of both cultivars and G2_2 gathering samples from the mature stage of ZD. Overall, the sample clustering pattern was clearly according to the leaf color phenotype.

### 2.3. Differentially Expressed Genes between the Two *L. indica* Cutlivars

**Figure 4:**
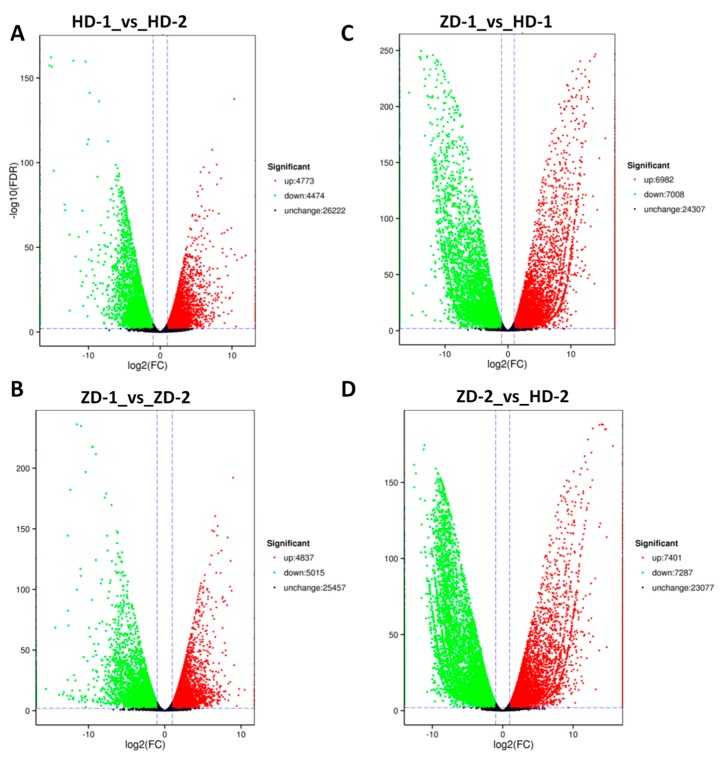
Volcano plot showing the up-regulated genes, down-regulated genes, and genes that were not regulated between pairs of compared samples. (**A**) HD-1_vs_HD-2, (**B**) ZD-1_vs_ZD-2; (**C**) ZD-1_vs_HD-1; and (**D**) ZD-2_vs_HD-2. HD represents the cultivar Lagerstroemia Dynamite, while ZD represents the cultivar Lagerstroemia Ebony Embers.

To uncover the genes involved in the different levels of anthocyanins in leaves of HD and ZD, the gene expression values expressed as FPKM were compared between cultivars and developmental stages. The differentially expressed genes (DEG) were detected with the following parameters: fold change >2 and a false discovery rate correction set at *p* < 0.01. The results showed large numbers of DEGs between compared pair of samples HD-1_vs_HD-2 (9247), ZD-1_vs_ZD-2 (9852), ZD-1_vs_HD-1 (13990), and ZD-2_vs_HD-2 (14688) (Figure 4). Gene ontology (GO) enrichment analysis was performed for these four types of DEGs (Figure S1). The metabolic process and cellular process were the most enriched GO terms in the biological process, the cell and cell part were the most enriched cellular component GO terms, while catalytic activity and binding were clearly enriched as molecular functions. These results suggest that transcription factors (binding activity) and high enzymatic activity are involved in the modulation of leaf coloration in *L. indica*. The highest numbers of DEGs (approximately 1/3 of total expressed genes) were observed by comparing the two cultivars independently of the stages, which indicates a large variation in their genetic make up. We focused our analysis on the genes related to the flavonoid–anthocyanin biosynthesis and MYB transcription factors detected within the DEGs.

### 2.4. DEGs Related to the Flavonoid–Anthocyanin Biosynthesis and Mechanisms Underlying the Differential Leaf Color Phenotypes

**Figure 5:**
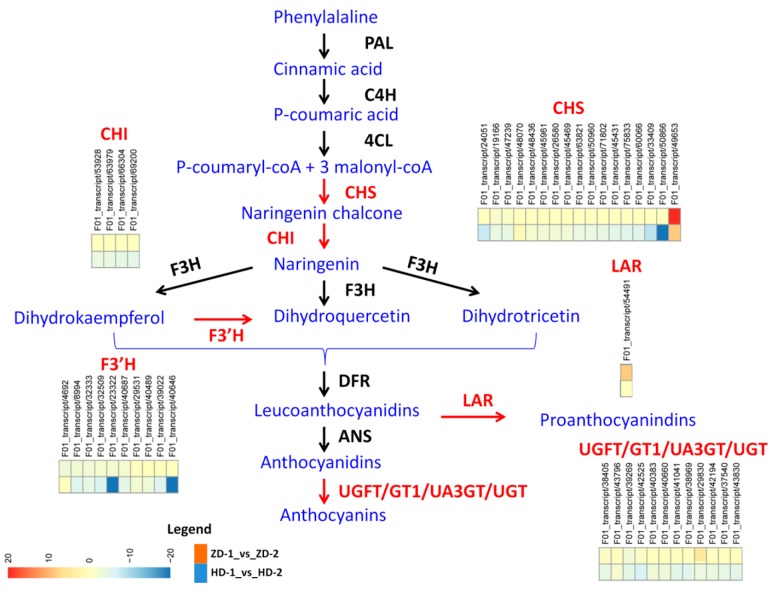
Flavonoid–anthocyanin biosynthetic genes in *L. indica*. The differentially expressed genes between HD and ZD from Stage 1 (young leaves) to Stage 2 (mature leaves) are highlighted in red color. Phenylalanine ammonia-lyase (PAL), cinnamic acid 4-hydroxylase (C4H), 4 coumarate CoA ligase (4CL), chalcone synthase (CHS), chalcone isomerase (CHI), flavanone 3-hydroxylase (F3H), flavonoid 3’-monooxygenase (F3′H), dihydroflavonol 4-reductase (DFR), by anthocyanidin synthase (ANS), leucoanthocyanidin reductase (LAR), UDP-flavonoid glucosyl transferase (UFGT), anthocyanidin 3-O-glucosyltransferase (UA3GT), anthocyanidin 5,3-O-glucosyltransferase (GT1), and anthocyanidin 3-O-glucoside 2′″-O-xylosyltransferase (UGT). The heatmap show the log2 fold change of the gene expression from Stage 1 to Stage 2. HD represents the cultivar Lagerstroemia Dynamite, while ZD represents the cultivar Lagerstroemia Ebony Embers.

Since we observed a higher content of total anthocyanins in the leaves of ZD than HD at the two developmental stages (Figure 1E), we further compared the expressed genes related to the flavonoid–anthocyanin biosynthesis between the two cultivars at each stage. We obtained 74 and 71 DEGs at Stage 1 and Stage 2, respectively, resulting in a total of 96 DEGs with the majority of these DEGs being higher expressed in ZD than HD, particularly at Stage 2 (Table S3). This result is not intriguing, and shows that a stronger activity of genes related to the flavonoid–anthocyanin biosynthesis in the leaves of ZD promotes a higher synthesis and accumulation of anthocyanins. To elucidate the molecular mechanism underlying the change in leaf color observed in HD while the leaf color of ZD remained stable, we investigated the DEGs between the two developmental stages in each cultivar. In total, 74 (10 up-regulated and 64 down-regulated) and 52 (nine up-regulated and 43 down-regulated) genes involved in the flavonoid–anthocyanin biosynthesis were differentially expressed from Stage 1 to Stage 2 in HD and ZD, respectively. The higher number of altered genes, particularly the down-regulated genes, in HD as compared to ZD highlights a mechanism to strongly limit anthocyanin biosynthesis. Thirty DEGs displayed the same pattern between the two cultivars, suggesting that they are not involved in the differential leaf color phenotype (Table S4). In contrast, 44 DEGs showed either opposite patterns between the two cultivars or a huge difference in the gene expression fold change from Stage 1 to Stage 2. We infer that these genes are crucial for the differential leaf color phenotype observed in the two cultivars. To better understand how these genes affect the leaf color, we mapped them on the flavonoid–anthocyanin biosynthesis pathways (Figure 5), which have been well characterized in plants [11,12]. The main precursors for flavonoids are 4-coumaroyl CoA and three molecules of malonyl CoA that produce chalcones by chalcone synthase (CHS) [13]. We identified 17 chalcone synthase [EC:2.3.1.74] (CHS) genes, including 16 strongly down-regulated in HD from Stage 1 to Stage 2, but these genes were unaltered or just slightly down-regulated in ZD. Flavanones are produced from chalcones via chalcone isomerase [EC:5.5.1.6] (CHI). We detected four CHI down-regulated in HD, but they were all unaffected in ZD at Stage 2. The pathway is further catalyzed by flavanone 3-hydroxylase [EC:1.14.11.9] (F3H) to yield dihydrokaempferol and subsequently by flavonoid 3’-monooxygenase [EC:1.14.14.82] (F3’H) to yield dihydroquercetin. We found 10 F3’H DEGs, nine of which were strongly silenced in HD from Stage 1 to Stage 2, but were unaffected or just slightly down-regulated in ZD. Dihydroflavonol 4-reductase (DFR) catalyzes the synthesis of leucoanthocyanidins, which could be converted into anthocyanidins (by anthocyanidin synthase [EC:1.14.20.4] (ANS)) or proanthocyanidins (by leucoanthocyanidin reductase [EC:1.17.1.3] (LAR)). In contrast to the previous flavonoid–anthocyanin biosynthetic DEGs, we found only one LAR (F01_transcript/54491) up-regulated in ZD but not affected in HD from Stage 1 to Stage 2, denoting a mechanism toward a high accumulation of proanthocyanidins in ZD leaves. Finally, anthocyanidins are converted into anthocyanins via UDP-flavonoid glucosyl transferase (UFGT) [13]. UFGT genes are active in the last step of anthocyanin modifications and without their actions, anthocyanins are unstable and can not accumulate in the cells to give the purple-red pigmentation [23]. In this study, we observed 12 various DEGs involved in this last step of anthocyanin modification, including two flavonol 3-O-glucosyltransferase [EC:2.4.1.91] (UFGT), seven anthocyanidin 3-O-glucosyltransferase [EC:2.4.1.115] (UA3GT), two anthocyanidin 5,3-O-glucosyltransferase [EC:2.4.1.-] (GT1), and one anthocyanidin 3-O-glucoside 2′″-O-xylosyltransferase [EC:2.4.2.51] (UGT). Interestingly, the expression levels of 11 out of these 12 genes were highly repressed from Stage 1 to Stage 2 in HD, while the majority was stably expressed in ZD. Distinctively, the gene F01_transcript/29830 (anthocyanidin 3-O-glucosyltransferase (UA3GT) was strongly up-regulated in ZD but was found to be repressed in HD. Collectively, our results demonstrate that in contrast to HD, ZD tends to keep a high activity level of key genes involved in the flavonoid–anthocyanin biosynthesis pathways throughout the leaf developmental stages in order to maintain the synthesis, accumulation, and modification of anthocyanins (probably proanthocyanidins, too).

### 2.5. Active MYB Transcripion Factors Regulating Gene Expression for the Differential Leaf Color Phenotypes

It has been documented in several plant species that the structural genes involved in the flavonoid–anthocyanin biosynthesis pathways are mainly regulated by MYB transcription factors [24]. In total, 663 and 620 TFs DEGs were involved in gene regulation activity from Stage 1 to Stage 2 in ZD and HD, respectively (Table S5). Among these TFs, we retrieved 61 and 60 MYB TFs in ZD and HD, respectively. Comparative analysis of the gene expression fold change of these MYB TFs showed that 36 MYBs were commonly differentially expressed in both cultivars with similar fold changes within each stage (Table S6). However, we uncovered 49 other MYBs genes, which exhibited contrasting expression patterns between the two cultivars and are likely to be the key regulators of the structural genes involved in the flavonoid–anthocyanin biosynthesis pathways in *L. indica* (Table S7).

**Figure 6:**
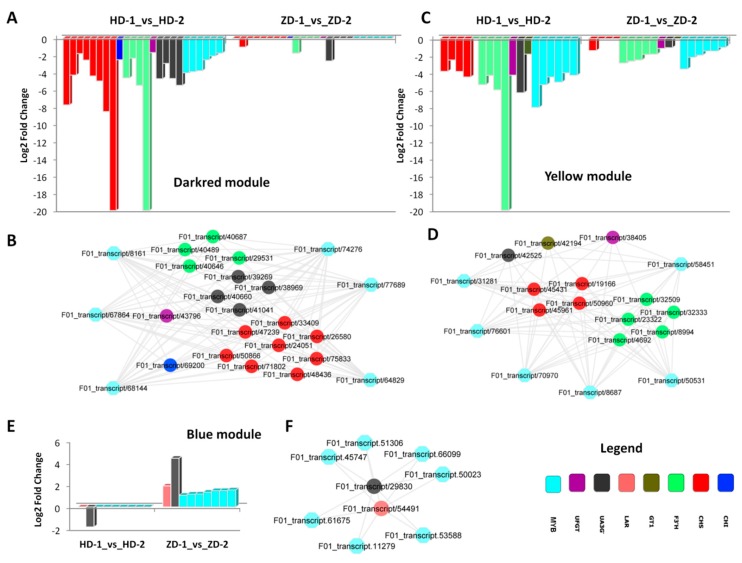
Gene co-expression analysis of the MYB regulators and their target genes related to the flavonoid–anthocyanin biosynthesis pathways in *L. indica*. (**A**,**B**) expression fold change and network of the genes co-expressed in the dark red module; (**B**,**C**) expression fold change and network of the genes co-expressed in the yellow module; (**D**,**E**) expression fold change and network of the genes co-expressed in the blue module. HD represents the cultivar Lagerstroemia Dynamite, while ZD represents the cultivar Lagerstroemia Ebony Embers.

The gene co-expression network approach constructs the network of genes (co-expressed modules) with co-activation across a group of samples. Genes with similar expression patterns under multiple, but resembling experimental conditions have a high probability of sharing similar functions or being involved in related biological pathways [25,26]. To better decipher the regulation pattern of the structural genes involved in the flavonoid–anthocyanin biosynthesis pathways by the candidate regulator MYBs, we performed a gene co-expression analysis [25]. To give more power to the gene co-expression analysis, we further sequenced the transcriptome from leaves of HD at two intermediate stages (IS-1 and IS-2) between Stage 1 and Stage 2, when the leaf color gradually changes form purple-red to green (Figure S2, Table S8). Gene co-expression analysis of a total of 18 RNA-seq data resulted into 22 co-expressed gene modules (Figure S3, Table S9). Interestingly, 19 key MYB regulators and 32 flavonoid–anthocyanin biosynthetic genes were co-expressed in three different modules: dark red, yellow, and blue. In each of these modules, the MYB transcription factors have a high probability of regulating the target co-expressed flavonoid–anthocyanin biosynthetic genes. In the dark red module, six MYB regulators are co-expressed with 18 structural genes, including CHS, CHI, F3′H, UFGT, and UA3G (Figure 6A,B). The MYBs were preferentially down-regulated in HD from Stage 1 to Stage 2, which correlated with the strong down-regulation of the target structural genes in HD. This suggests that MYBs from this module are positive modulators of color formation in leaves of *L. indica*. Similarly in the yellow module, six MYBs were strongly down-regulated in HD as compared to ZD, and this correlated with a more reduced expression level of the structural genes (CHS, F3′H, UFGT, GT1, and UA3G) in HD (Figure 6C,D). Finally, in the blue module, seven MYBs were preferentially up-regulated in ZD to induce the expression levels of one LAR and one UA3G gene (Figure 6E,F). Globally, the gene co-expression analysis revealed that MYBs are positive modulators of the structural genes and the strong down-regulation of most of these MYB regulators from Stage 1 to Stage 2 observed in HD may limit the activity of the enzymes that catalyze the flavonoid–anthocyanin biosynthesis pathways, resulting in a reduced anthocyanin accumulation in the leaves.

To confirm the differential expression levels of the candidate structural genes and the co-expressed MYB transcription factors detected by the RNA-seq analysis, we conducted a quantitative real-time PCR on 21 selected genes from all modules. As expected, the qRT-PCR results were well correlated with the RNA-seq report (*R*^2^ = 0.84; Figure S4), demonstrating the reliability of the report from this study.

## 3. Discussion

Although it is well known that anthocyanins are the key pigments coloring plant organs [10], their composition and concentration greatly vary among plant species [27]; therefore, it is impossible to predict the key molecules underlying specific colorations in plants without a detailed metabolic profiling. Anthocyanidins are the aglycone units of anthocyanins, and there are six major types found widely in plants, namely pelargonidin, cyanidin, peonidin, delphinidin, petunidin, and malvidin [28]. There was no previous report of the leaf anthocyanin profile in *L. indica*, but several studies were conducted on the flowers of different cultivars. Collectively, four anthocyanins were reported in *L. indica* flowers, including delphinidin 3-O-glucoside, petunidin 3-Oglucoside, cyanidin 3-O-glucoside, and malvidin 3-O-glucoside [29,30,31,32]. It is worth mentioning that these authors analyzed flowers with various colors, including purple-red, purple, purple-violet, violet, and white. In the present study, we obtained a less diverse set of anthocyanins, and only two of the flower anthocyanins (delphinidin 3-O-glucoside and cyanidin 3-O-glucoside) were detected in the leaves. This is understandable since only one leaf color was studied here. The intriguing findings in this study are those regarding the diversity of cyanidin glycoside-derived and methylated-derived compounds in both cultivars (cyanidin O-syringic acid, cyanidin 3-O-glucoside, cyanidin 3,5-O-diglucoside, and rosinidin O-hexoside), although cyanidin was only found in HD. The absence of cyanidin in ZD implies that it is systematically converted into the glycoside-derived and methylated-derived forms. Pelargonidin and cyanidin are the red series pigments in plants [29]. The absence of pelargonidin in the leaves of both cultivars indicate that cyanidin derivatives represent the main molecules conferring the purple-red coloration. Our results are in agreement with the previous report of Zhang et al. [32], who showed that cyanidin 3-O-glucoside was mainly concentrated in cultivars with purple-red flowers.

RNA sequencing offers the opportunity to simultaneously profile the expression levels of thousand of genes [33]. Zhang et al. [34] and Wang et al. [35] sequenced the leaf transcriptome in *L. indica* to study the flowering regulatory genes and powdery mildew disease responsive genes, respectively. Globally, these authors assembled ~37000 genes, which is lower than the number of unigenes reported in the present study (45925). The difference in the numbers of detected genes may be attributed to the advanced sequencing platform and bioinformatic packages employed for unigene assembly in this work. Our goal was to explore the molecular mechanism underlying the differential leaf color phenotypes in the two cutivars, with a focus on the genes involved in the biosynthetic pathway of anthocyanins and their regulators [11,12]. In fact, the quantitative and qualitative variation of anthocyanins in plants are strongly correlated with the differential expression of key structural genes involved in the anthocyanin biosynthesis pathways [18,36]. In this study, several classes of structural genes related to the flavonoid–anthocyanin biosynthesis were differentially expressed between the two cultivars and have been mapped to the early steps (chalcone synthase (CHS), chalcone isomerase (CHI), and flavonoid 3’-monooxygenase (F3′H)) and late steps (leucoanthocyanidin reductase (LAR), UDP-flavonoid glucosyl transferase (UFGT), anthocyanidin 3-O-glucosyltransferase (UA3GT), anthocyanidin 5,3-O-glucosyltransferase (GT1), and anthocyanidin 3-O-glucoside 2′″-O-xylosyltransferase (UGT)) (Figure 5) [12]. These genes were globally down-regulated from the young leaf stage to the mature stage in both cultivars; however, we noticed that ZD tends to maintain a stronger activity as compared to HD (Table S3), which presumably favors the observed high accumulation of anthocyanins in ZD (Figure 1E). More often, genes belonging to either the early steps or the late steps, but not both simultaneously have been reported to differentially modulate anthocyanin contents in contrasting colored samples. For example, Chen et al. [37] demonstrated that low expression levels of C4H, CHS, and F3H in white petals, contrarily to the red petals of peach, reduce the formation of dihydro-kaempferol (DHK), and thereby inhibit the anthocyanin accumulation. In addition, Jiao et al. [38] showed that PAL was weakly expressed in the white-flesh peach and limits anthocyanin production. In contrast, Zhuang et al. [21] showed that a strong anthocyanin accumulation in purple turnip was attributed to an up-regulation of DFR, ANS, and UFGT genes. LAR converts leucoanthocyanidins into proanthocyanidins. In this study, the up-regulation of the LAR gene (*F01_transcript/54491*) in ZD suggests an increment of the proanthocyanidins content from Stage 1 to Stage 2, but this mechanism may not be relevant to the stable leaf coloration observed in ZD, since proanthocyanidins are colorless in nature [39]. The class of genes involved in the modification of anthocyanidins (UDP-flavonoid glucosyl transferase (UFGT), anthocyanidin 3-O-glucosyltransferase (UA3GT), anthocyanidin 5,3-O-glucosyltransferase (GT1), and anthocyanidin 3-O-glucoside 2′″-O-xylosyltransferase (UGT)) was particularly enriched (Figure 5). This was expected, since the anthocyanins detected in leaf samples were mainly glycoside-derived compounds (Table S1). Anthocyanidins are highly unstable and easily susceptible to degradation; therefore, glycosylation is essential to stabilize them [40]. Furthermore, glycosylation serve as a signal for transport of the anthocyanins to vacuoles, where they can function as pigments [41]. Since most of these genes were higher expressed in ZD than HD, correlating with the stronger content of glycoside-derived anthocyanins, we deduce that the glycosylation of anthocyanins (particularly cyanidin) is a key mechanism for the stable purple-red colored leaf phenotype observed in ZD, exactly as previously demonstrated in peach [42].

The expression levels of structural genes involved in the flavonoid–anthocyanin pathway are in part regulated by transcription factors (TF), particularly by the MYB family members [24]. We uncovered 49 candidate MYBs that are likely to be the key regulators of the structural genes involved in the flavonoid–anthocyanin pathway (Table S7). Many studies have reported several differentially expressed MYB genes as potential regulators, but the target genes of each specific MYB regulator and the regulatory network are often overlooked. The mechanisms of gene expression regulation by a TF could be simple (direct binding to the binding motif in the promoter region of the targets) or more complex, involving other cofactors. An example of the complex regulation mechanism is the feed-forward loop mechanism where a three-gene pattern is composed of two input transcription factors, one of which regulates the other, which both jointly regulate a target gene [43]. Hence, it is essential to clearly delineate the network of interaction between candidate MYBs and their targets in order to facilitate the directional manipulation of the expression levels of the structural genes involved in the flavonoid–anthocyanin pathway. In this study, we revealed three co-expressed modules containing candidate MYB regulators and their target structural genes (Figure 6). Overall, we found that MYBs are positive regulators of these structural genes; therefore, increasing the activity levels of some MYBs from these co-expressed modules, particularly those from the dark red and yellow modules, may have high potential to confer stable purple-red coloration in the leaves of HD and other *L. indica* cultivars.

## 4. Materials and Methods

### 4.1. Plant Materials

Two cultivars of *Lagerstroemia indica* L. were used as plant materials. The cultivar Lagerstroemia Dynamite was developed by the Carl Whitcomb breeding program, (Carl Whitcomb Lacebark Inc. Stillwater, OK, USA) and features purple-red young leaves, which gradually turn into a green color when they mature (Figure 1, Figure S2). The second cultivar, Lagerstroemia Ebony Embers released by the USDA, displays stable purple-red leaves throughout all the leaf developmental stages. In this study, Lagerstroemia Dynamite and Lagerstroemia Ebony Embers were named as HD and ZD, respectively. Both cultivars were grown under natural environmental conditions in the experimental station of the Hunan Academy of Forestry, China. Leaf blades were collected at different developmental stages (Figure 1, Figure S2) from three independent plants (2 years old) of each cultivar, quickly frozen in liquid nitrogen, and stored at −80 °C until further use.

### 4.2. Anthocyanin Analysis

The sample preparation, extract analysis, anthocyanin identification and quantification were performed at Wuhan MetWare Biotechnology Co., Ltd. (www.metware.cn) following their standard procedures and previously described by Cao et al. [22]. Before the data analysis, quality control (QC) analysis was conducted to confirm the reliability of the data. The QC sample was prepared by the mixture of sample extracts and inserted into every four samples to monitor the changes in repeated analyses. Data matrices with the intensity of the metabolite features from the samples were uploaded to the Analyst 1.6.1 software (AB SCIEX, Ontario, Canada) for statistical analyses. The supervised multivariate method, partial least squares-discriminant analysis (PLS-DA), was used to maximize the metabolome differences between the pair of samples. The relative importance of each metabolite to the PLS-DA model was checked using the parameter called variable importance in projection (VIP). Metabolites with VIP ≥1 and fold change ≥2 or fold change ≤0.5 were considered as differential metabolites for group discrimination [22].

### 4.3. Transcriptome Sequencing and Data Analysis

RNA extraction, transcriptome library preparation, sequencing, and bioinformatics analysis were conducted at the Biomarker Technologies (Beijing, China, www.biomarker.com.cn) following their standard procedures and previously described by Zhu et al. [44]. Briefly, total RNA was extracted from the leaf samples using a Spin Column Plant total RNA Purification Kit (Sangon Biotech, Shanghai, China) according to the manufacturer’s instructions. Sequencing libraries were constructed following the protocol of the Gene Expression Sample Prep Kit (Illumina, San Diego, CA, USA). The first-strand cDNAs were synthesized from the total RNA with random hexamer primers, followed by second-strand cDNAs synthesis using DNA polymerase I (New England BioLabs, Ipswich, MA, USA) and RNase H (Invitrogen, Waltham, MA, USA). After end repair, adaptor ligation, and the addition of index codes for each sample, PCR amplification was conducted. The purity and quality of the libraries were measured by an Agilent 2100 Bioanalyzer (Agilent Technologies, Santa Clara, CA, USA) and Qubit 2.0 (Life Technologies, Carlsbad, CA, USA). Then, the libraries were pair-end sequenced by using the Illumina HiSeq 2500 platform (Illumina Inc., San Diego, USA).

The raw RNA-seq reads were quality-checked with the FastQC package (http://www.bioinformatics.babraham.ac.uk/projects/fastqc/), and adaptor sequences and low-quality reads (containing >50% bases with a Phred quality score <15 and reads with more than 1% ambiguous residues N) were removed. The high-quality reads from all the libraries were de novo assembled into transcripts using the software Trinity (version r20140717, [45]) by employing the paired-end method. Next, the transcripts were realigned to construct unigenes. The assembled unigenes were annotated by searching against various databases such as the Kyoto Encyclopedia of Genes and Genomes (KEGG) [46], Gene Ontology (GO) [47], Clusters of Orthologous Groups (COG) [48], PfAM [49], Swiss-Prot [50], eggNOG [51], NR [52], and euKaryotic Orthologous Groups (KOG) [53] using BLAST [54] with a threshold of E-value <1.0 × 10^−5^. The software KOBAS2.0 [55] was employed to get the unigene KEGG orthology. The analogs of the unigene amino acid sequences were searched against the Pfam database [48] using the HMMER tool [56] with a threshold of E-value <1.0 × 10^−10^. The unigenes were counted and normalized into fragments per kilobase of transcript per million fragments mapped reads (FPKM) value using RSEM [57]. Differentially expressed genes (DEGs) between pairs of samples were determined using the EdgeR Bioconductor package [58]. False discovery rate values less than 0.01 and |fold change|≥2 were set as criteria to decide the significant differences in gene expression.

### 4.4. Gene Co-Expression Analysis

Weighted Gene Co-Expression Network Analysis (WGCNA) package version 1.61 [59] was used to construct the gene co-expression networks from the normalized log2-transformed FPKM matrix as described by Lv et al. [25] and Dossa et al. [60]. Network visualization for the co-expressed gene modules related to MYB and flavonoid–anthocyanin biosynthesis pathways was performed using the Cytoscape software version 3.6.1 [61].

### 4.5. Quantitative RT-PCR Analysis

Quantitative PCR was performed using the SYBR Premix Ex Taq™ Kit (Takara, Dalian, China) according to the manufacturer’s instructions on the StepOne plus Real time PCR Platform (Applied Biosystems, CA, USA) with the following protocol: 95 °C for 10 min, followed by 40 cycles of 95 °C for 15 s, and at 60 °C for 60 s [62]. Each reaction was performed using a 20-μL mixture containing 10 μL of 2 × ChamQ SYBR qPCR Master Mix, 6 μL of nuclease-free water, 1 μL of each primer (10 mM), and 2 μL of four-fold diluted cDNA. All of the reactions were run in 96-well plates, and each cDNA was analyzed in triplicate. Specific primer pairs of 21 selected genes were designed using the Primer Premier 5.0 [63] (Table S10). The *Actin* gene was used as the internal control. Data are presented as relative transcript levels based on the 2^−∆∆Ct^ method [64].

## Figures and Tables

**Table 1:** Concentration of the anthocyanins detected in the two *L. indica* cutlivars and their log2 fold-change values. Bold values are those significantly changed between compared samples.

Compounds	HD-1	ZD-1	HD-2	ZD-2	Log2 Fold Change			
					HD-1_vs_HD-2	ZD-1_vs_ZD-2	ZD-1_vs_HD-1	ZD-2_vs_HD-2
Peonidin O-hexoside	24257333.3	13935333.3	2037100	12121666.7	**−3.57**	−0.20	0.80	**−2.57**
Rosinidin O-hexoside	89211.3333	144180	0	43131	**−13.28**	**−1.74**	−0.69	**−12.23**
Cyanidin O-syringic acid	2205133.33	4702233.33	144803.333	2828266.67	**−3.93**	−0.73	**−1.09**	**−4.29**
Cyanidin 3-O-glucoside	7512833.33	17313666.7	443633.333	8068766.67	**−4.08**	**−1.10**	**−1.20**	**−4.18**
Delphinidin 3-O-glucoside	3606100	5734733.33	2009400	5926300	–0.84	0.05	−0.67	**−1.56**
Cyanidin 3,5-O-diglucoside	4405800	13112000	394216.667	7217400	**−3.48**	−0.86	**−1.57**	**−4.19**
Cyanidin	801780	0	484516.667	0	−0.73	0.00	**16.44**	**15.72**

**Table 2:** Overview of the transcriptome sequencing dataset and quality check.

SampleID.	Raw Read Number	Base Number	GC (%)	Q20 (%)	Q30 (%)
HD-1_1	29963667	8930322034	51.21	97.91	94.18
HD-2_1	29373060	8751104486	51.26	97.85	94.09
HD-2_2	20168830	6023966824	50.7	97.82	93.98
HD-2_3	23631048	7060390512	50.81	97.83	94
HD-1_2	23919615	7129091888	51.23	98	94.41
HD-1_2	23467265	7003557242	50.74	97.88	94.23
ZD-1_1	24273602	7232878206	51.32	97.69	93.8
ZD-1_2	21891679	6530271554	51.24	97.97	94.22
ZD-1_3	22505602	6715319880	51.25	97.88	94.12
ZD-2_1	21339681	6361383236	51.24	97.85	93.99
ZD-2_2	21255491	6336179806	51.32	97.8	93.93
ZD-2_3	22210402	6625694066	51.27	97.88	94.04

## Supplementary Materials

Supplementary materials can be found at https://www.mdpi.com/1422-0067/20/22/5636/s1. Table S1. Quantification of the detected anthocyanins in the two *L. indica* cultivars at Stage 1 (young leaves) and Stage 2 (mature leaves); Table S2. Genes encoding transcription factors detected in *L. indica* transcriptome; Table S3. List of the differentially expressed genes between ZD and HD at Stage 1 and Stage 2 and their log2 fold change values; Table S4. List of the differentially expressed genes between Stage 1 and Stage 2 in HD and ZD that display the same fold-change patterns between the two cultivars; Table S5. Differentially expressed genes encoding transcription factors form Stage 1 to Stage 2; Table S6. List of the differentially expressed MYB genes between Stage 1 and Stage 2 in HD and ZD that display the same fold-change patterns between the two cultivars; Table S7. List of the differentially expressed MYB genes between Stage 1 and Stage 2 in HD and ZD that display different patterns of fold change of a huge difference between the two cultivars; Table S8. Overview of the transcriptome sequencing dataset and quality check of the leaves from HD at the intermediate stage (IS1 and IS2); Table S9. List of the genes belonging of each of the 22 modules detected through gene co-expression analysis; Table S10. The primer sequences of genes used for quantitative real time PCR; Figure S1. Gene ontology enrichment analysis of the differentially expressed genes between (A) HD-1_vs_HD-2, (B) ZD-1_vs_ZD-2; (C) ZD-1_vs_HD-1; (D) ZD-2_vs_HD-2. HD represents the cultivar Lagerstroemia Dynamite, while ZD represents the cultivar Lagerstroemia Ebony Embers. Figure S2. The phenotypes of HD leaves at the intermediate Stage 1 and Stage 2 when the leaf color is gradually turning from purple-red to green; Figure S3. Dendrogram clustering of the genes and identification of the co-expressed modules; Figure S4. qRT-PCR (2^−ΔΔ*C*t^) analysis of 21 selected genes within the differentially expressed genes detected in this study. Correlation analysis between qRT-PCR and RNA-seq (log2 fold change).

## References

[1] Pounders C., Rinehart T., Edwards N., Knight P. (2007). An analysis of combining ability for height, leaf out, bloom date, and flower color for crapemyrtle. HortScience.

[2] Liu Y.S., Zetter R., Ferguson D.K., Zou C. (2008). Lagerstroemia (Lythraceae) pollen from the Miocene of eastern China. Grana.

[3] Cabrera R.I. Evaluating and promoting the cosmopolitan and multipurpose Lagerstroemia. Proceedings of the XXVI International Horticultural Congress.

[4] Pounders C., Scheffler B.E., Rinehart T.A. (2013). ‘Ebony Embers’, ‘Ebony Fire’, ‘Ebony Flame’, ‘EbonyGlow’, and ‘Ebony and Ivory’ Dark-leaf Crapemyrtles. HortScience.

[5] Zhengkang P. (2006). Cultivation Managements of *Lagerstroemia indica* and its Application in the Landscape. J. Anhui Agric. Sci..

[6] Knox G. (2014). New Crapemyrtles with Burgundy Leaves from Spring through Fall. http://nwdistrict.ifas.ufl.edu/hort/2014/07/21/new-crapemyrtles-with-burgundy-leaves-from-spring-through-fall/.

[7] Pounders C.T., Blythe E.K., Fare D.C., Knox G.W., Sibley J.L. (2010). Crapemyrtle genotype × environment interactions, and trait stability for plant height, leaf-out, and flowering. HortScience.

[8] Gilman E.F., Watson D.G., Klein R.W., Koeser A.K., Hilbert D.R., McLean D.C. (2018). *Lagerstroemia indica*: Crapemyrtle. https://edis.ifas.ufl.edu.

[9] Mohan Jain S., Brar D.S. (2010). Molecular Techniques in Crop Improvement.

[10] Chen C. (2015). Overview of plant pigments. Pigments in Fruits and Vegetables.

[11] Tanaka Y., Ohmiya A. (2008). Seeing is believing: Engineering anthocyanin and carotenoid biosynthetic pathways. Curr. Opin. Biotechnol..

[12] Jaakola L. (2013). New insights into the regulation of anthocyanin biosynthesis in fruits. Trends Plant Sci..

[13] Dixon R.A., Steele C.L. (1999). Flavonoids and isoflavonoids—A gold mine for metabolic engineering. Trends Plant Sci..

[14] Moyano E., Martinez-Garcia J.F., Martin C. (1996). Apparent redundancy in myb gene function provides gearing for the control of flavonoid biosynthesis in antirrhinum flowers. Plant Cell.

[15] To K.Y., Wang C.K., Silva T.D. (2006). Molecular breeding of flower color. Floriculture Ornamental and Plant Biotechnology: Advances and Topical Issues Volume I.

[16] Li Z., Zhao M., Jin J., Zhao L., Xu Z. (2018). Anthocyanins and their biosynthetic genes in three novel-colored Rosa rugosa cultivars and their parents. Plant Physiol. Biochem..

[17] Jiang G., Li Z., Song Y., Zhu H., Lin S., Huang R., Jiang Y., Duan X. (2019). LcNAC13 Physically Interacts with LcR1MYB1 to Coregulate Anthocyanin Biosynthesis-Related Genes during Litchi Fruit Ripening. Biomolecules.

[18] Lloyd A., Brockman A., Aguirre L., Campbell A., Bean A., Cantero A., Gonzalez A. (2017). Advances in the MYB–bHLH–WD repeat (MBW) pigment regulatory model: Addition of a WRKY factor and co-option of an anthocyanin MYB for betalain regulation. Plant Cell Physiol..

[19] Zhou H., Lin-Wang K., Wang H., Gu C., Dare A.P., Espley R.V., He H., Allan A.C., Han Y. (2015). Molecular genetics of blood-fleshed peach reveals activation of anthocyanin biosynthesis by NAC transcription factors. Plant J..

[20] He L., Tang R., Shi X., Wang W., Cao Q., Liu X., Wang T., Sun Y., Zhang H., Li R. (2019). Uncovering anthocyanin biosynthesis related microRNAs and their target genes by small RNA and degradome sequencing in tuberous roots of sweet potato. BMC Plant Biol..

[21] Zhuang H., Lou Q., Liu H., Han H., Wang Q., Tang Z., Ma Y., Wang H. (2019). Differential Regulation of Anthocyanins in Green and Purple Turnips Revealed by Combined De Novo Transcriptome and Metabolome Analysis. Int. J. Mol. Sci..

[22] Cao H., Ji Y., Li S., Lu L., Tian M., Yang W., Li H. (2019). Extensive Metabolic Profiles of Leaves and Stems from the Medicinal Plant Dendrobium officinale Kimura et Migo. Metabolites.

[23] Fosket D.E., Donald E. (1994). The Genetic Basis of Plant Development. Fosket, Plant Growth and Development.

[24] Allan A.C., Hellens R.P., Laing W.A. (2008). MYB transcription factors that colour our fruit. Trends Plant Sci..

[25] Lv Y., Xu L., Dossa K., Zhou K., Zhu M., Xie H., Tang S., Yu Y., Guo X., Zhou B. (2019). Identification of putative drought-responsive genes in rice using gene co-expression analysis. Bioinformation.

[26] Lee H.K., Hsu A.K., Sajdak J., Qin J., Pavlidis P. (2004). Overexpression analysis of human genes across many microarray data sets. Genome Res..

[27] Veberic R., Slatnar A., Bizjak J., Stampar F., Mikulic-Petkovsek M. (2015). Anthocyanin composition of different wild and cultivated berry species. LWT Food Sci. Technol..

[28] Kong J.M., Chia L.S., Goh N.K., Chia T.F., Brouillard R. (2003). Analysis and biological activities of anthocyanins. Phytochemistry.

[29] Egolf D.R., Santamour F.S. (1975). Anthocyanin pigments and breeding potential in crape myrtle (*Lagerstreomia indica* L.) and rose of Sharon (Hibiscus syriacus L.). HortScience.

[30] Toki K. (1989). Anthocyanin pigments and breeding potential of blue flowers in *Lagerstreomia indica*. BioHort.

[31] Toki K., Katsuyama N. (1995). Pigments and color variation in flowers of *Lagerstroemia indica*. J. Jpn. Soc. Hortic. Sci..

[32] Zhang J., Wang L.-S., Gao J.-M., Shu Q.-Y., Li C.-H., Yao J., Hao Q., Zhang J.-J. (2008). Determination of Anthocyanins and Exploration of Relationship between Their Composition and Petal Coloration in Crape Myrtle (*Lagerstroemia* hybrid). J. Integr. Plant Biol..

[33] Van Dam S., Craig T., de Magalhães J.P. (2015). GeneFriends: A human RNA-seq-based gene and transcript co-expression database. Nucleic Acids Res..

[34] Zhang Z.Y., Wang P., Li Y., Ma L.L., Li L.F., Yang R.T., Ma Y.Z., Wang S., Wang Q. (2014). Global transcriptome analysis and identification of the flowering regulatory genes expressed in leaves of *Lagerstroemia indica*. DNA Cell Biol..

[35] Wang X., Shi W., Rinehart T. (2015). Transcriptomes That Confer to Plant Defense against Powdery Mildew Disease in *Lagerstroemia indica*. Int. J. Genom..

[36] Naing A.H., Kim C.K. (2018). Roles of R2R3-MYB transcription factors in transcriptional regulation of anthocyanin biosynthesis in horticultural plants. Plant Mol. Biol..

[37] Chen Y., Mao Y., Liu H., Yu F., Li S., Yin T. (2014). Transcriptome analysis of differentially expressed genes relevant to variegation in peach flowers. PLoS ONE.

[38] Jiao Y., Ma R.J., Shen Z.J., Yan J., Yu M.L. (2014). Gene regulation of anthocyanin biosynthesis in two blood-flesh peach (*Prunus persica* (L) Batsch) cultivars during fruit development. J. Zhejiang Univ. Sci. B.

[39] He F., Pan Q.H., She Y., Duan C.Q. (2008). Biosynthesis and genetic regulation of proanthocyanidins in plants. Molecules.

[40] Hellström J., Mattila P., Karjalainen R.O. (2013). Stability of anthocyanins in berry juices stored at different temperatures. J. Food Compos. Anal..

[41] Ono E., Homma Y., Horikawa M., Kunikane-Doi S., Imai H., Takahashi S., Kawai Y., Ishiguro M., Fukui Y., Nakayama T. (2010). Functional differentiation of the glycosyltransferases that contribute to the chemical diversity of bioactive flavonol glycosides in grapevines (Vitis vinifera). Plant Cell.

[42] Cheng J., Wei G., Zhou H., Gu C., Vimolmangkang S., Liao L., Han Y.P. (2014). Unraveling the mechanism underlying the glycosylation and methylation of anthocyanins in peach. Plant Physiol..

[43] Mangan S., Alon U. (2003). Structure and function of the feed-forward loop network motif. Proc. Natl. Acad. Sci. USA.

[44] Zhu C., Li X., Zheng J. (2018). Transcriptome profiling using Illumina- and SMRT-based RNAseq of hot pepper for in-depth understanding of genes involved in CMV infection. Gene.

[45] Grabherr M.G., Haas B.J., Yassour M., Levin J.Z., Thompson D.A., Amit I., Adiconis X., Fan L., Raychowdhury R., Zeng Q. (2011). Full length transcriptome assembly from RNA Seq data without a reference genome. Nat. Biotechnol..

[46] Kanehisa M., Goto S., Kawashima S., Okuno Y., Hattori M. (2004). The KEGG resource for deciphering the genome. Nucleic Acids Res..

[47] Ashburner M., Ball C.A., Blake J.A., Botstein D., Butler H., Cherry J.M., Davis A.P., Dolinski K., Dwight S.S., Eppig J.T. (2000). Gene ontology: Tool for the unification of biology. Nat. Genet..

[48] Tatusov R.L., Galperin M.Y., Natale D.A. (2000). The COG database: A tool for genome scale analysis of protein functions and evolution. Nucleic Acids Res..

[49] Finn R.D., Bateman A., Clements J., Coggill P., Eberhardt R.Y., Eddy S.R., Heger A., Hetherington K., Holm L., Mistry J. (2013). Pfam: The protein families database. Nucleic Acids Res..

[50] Apweiler R., Bairoch A., Wu C.H., Barker W.C., Boeckmann B., Ferro S., Gasteiger E., Huang H., Lopez R., Magrane M. (2004). UniProt: The Universal Protein knowledgebase. Nucleic Acids Res..

[51] Huerta-Cepas J., Szklarczyk D., Forslund K., Cook H., Heller D., Walter M.C., Rattei T., Mende D.R., Sunagawa S., Kuhn M. (2015). eggNOG 4.5: A hierarchical orthology framework with improved functional annotations for eukaryotic, prokaryotic and viral sequences. Nucleic Acids Res..

[52] Deng Y.Y., Li J.Q., Wu S.F., Zhu Y.P., Chen Y.W., He F.C. (2006). Integrated nr Database in Protein Annotation System and Its Localization. Comput. Eng..

[53] Koonin E.V., Fedorova N.D., Jackson J.D., Jacobs A.R., Krylov D.M., Makarova K.S., Mazumder R., Mekhedov S.L., Nikolskaya A.N., Rao B.S. (2004). A comprehensive evolutionary classification of proteins encoded in complete eukaryotic genomes. Genome Biol..

[54] Altschul S.F., Madden T.L., Schäffer A.A., Zhang J., Zhang Z., Miller W., Lipman D.J. (1997). Gapped BLAST and PSI BLAST: A New Generation of Protein Database Search Programs. Nucleic Acids Res..

[55] Xie C., Mao X., Huang J., Ding Y., Wu J., Dong S., Kong L., Gao G., Li C., Wei L. (2011). KOBAS 2.0: A web server for annotation and identification of enriched pathways and diseases. Nucleic Acids Res..

[56] Eddy S.R. (1998). Profile hidden Markov models. Bioinformatics.

[57] Li B., Colin N.D. (2011). RSEM: Accurate transcript quantification from RNA Seq data with or without a reference genome. BMC Bioinform..

[58] Robinson M.D., McCarthy D.J., Smyth G.K. (2010). edgeR: A Bioconductor package for differential expression analysis of digital gene expression data. Bioinformatics.

[59] Su G., Morris J.H., Demchak B., Bader G.D. (2014). Biological network exploration with Cytoscape 3. Curr. Protoc. Bioinform..

[60] Dossa K., Mmadi M.A., Zhou R., Zhang T., Su R., Zhang Y., Wang L., You J., Zhang X. (2019). Depicting the Core Transcriptome Modulating Multiple Abiotic Stresses Responses in Sesame (*Sesamum indicum* L.). Int. J. Mol. Sci..

[61] Langfelder P., Horvath S. (2008). WGCNA: An R package for weighted correlation network analysis. BMC Bioinform..

[62] Dossa K., Mmadi M.A., Zhou R., Zhou Q., Yang M., Cisse N., Diouf D., Wang L., Zhang X. (2018). The contrasting response to drought and waterlogging is underpinned by divergent DNA methylation programs associated with transcript accumulation in sesame. Plant Sci..

[63] Lalitha S. (2000). Primer premier 5. Biotechnol. Softw. Internet Rep..

[64] Livak K.J., Schmittgen T.D. (2001). Analysis of relative gene expression data using real-time quantitative PCR and the 2^−ΔΔ*C*^*^t^* method. Methods.

