# Supplementary figures and images for: Exploring the Molecular Mechanism underlying the Stable Purple-Red Leaf Phenotype in Lagerstroemia indica cv. Ebony Embers
Source: Int J Mol Sci. 2019 Nov 11;20(22):5636. doi: 10.3390/ijms20225636 (PMC6888693; doi:10.3390/ijms20225636)

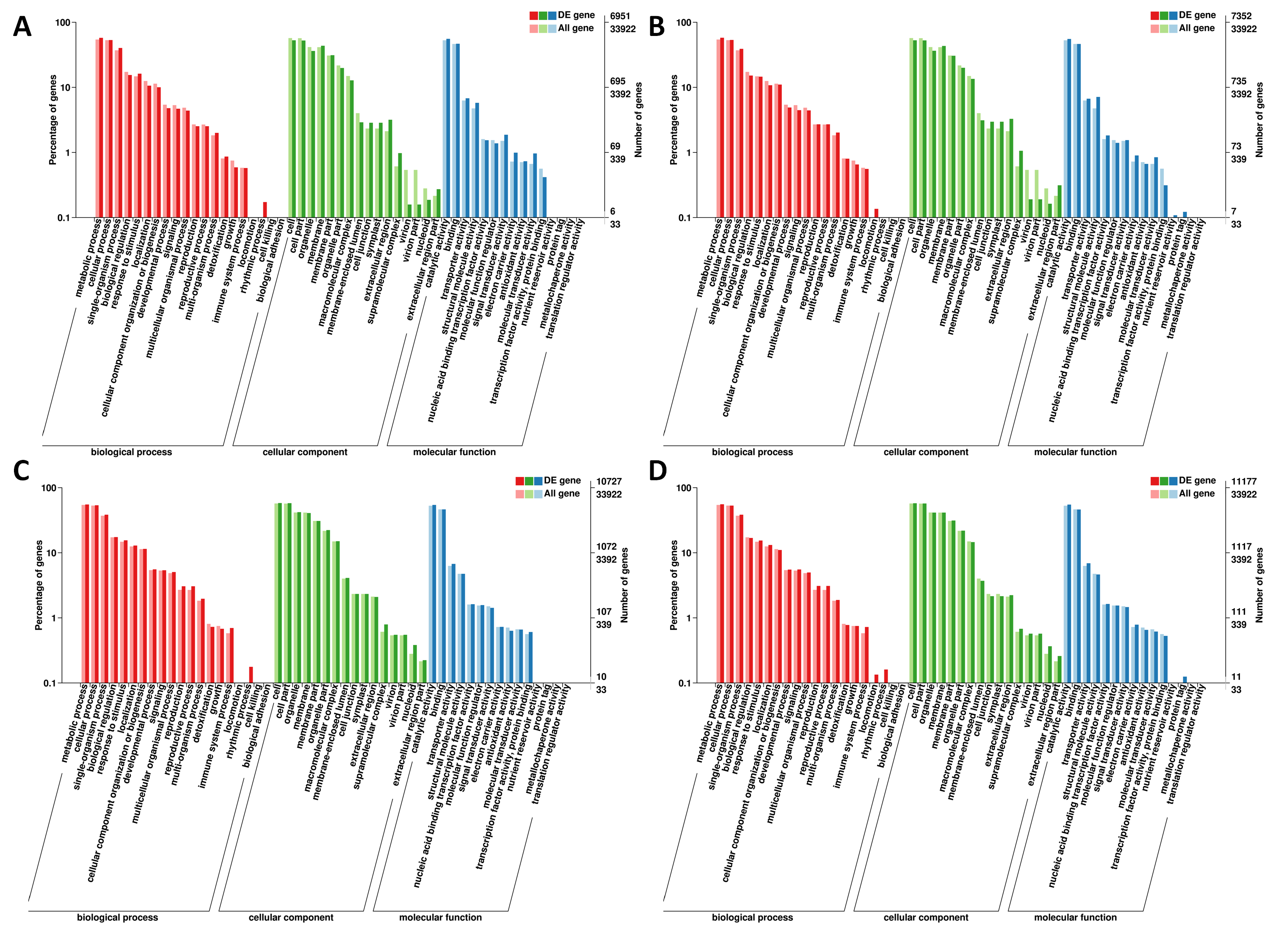

Supplement: Supplementary file 1 [file ijms-20-05636-s001.zip › Figure S1.tif]

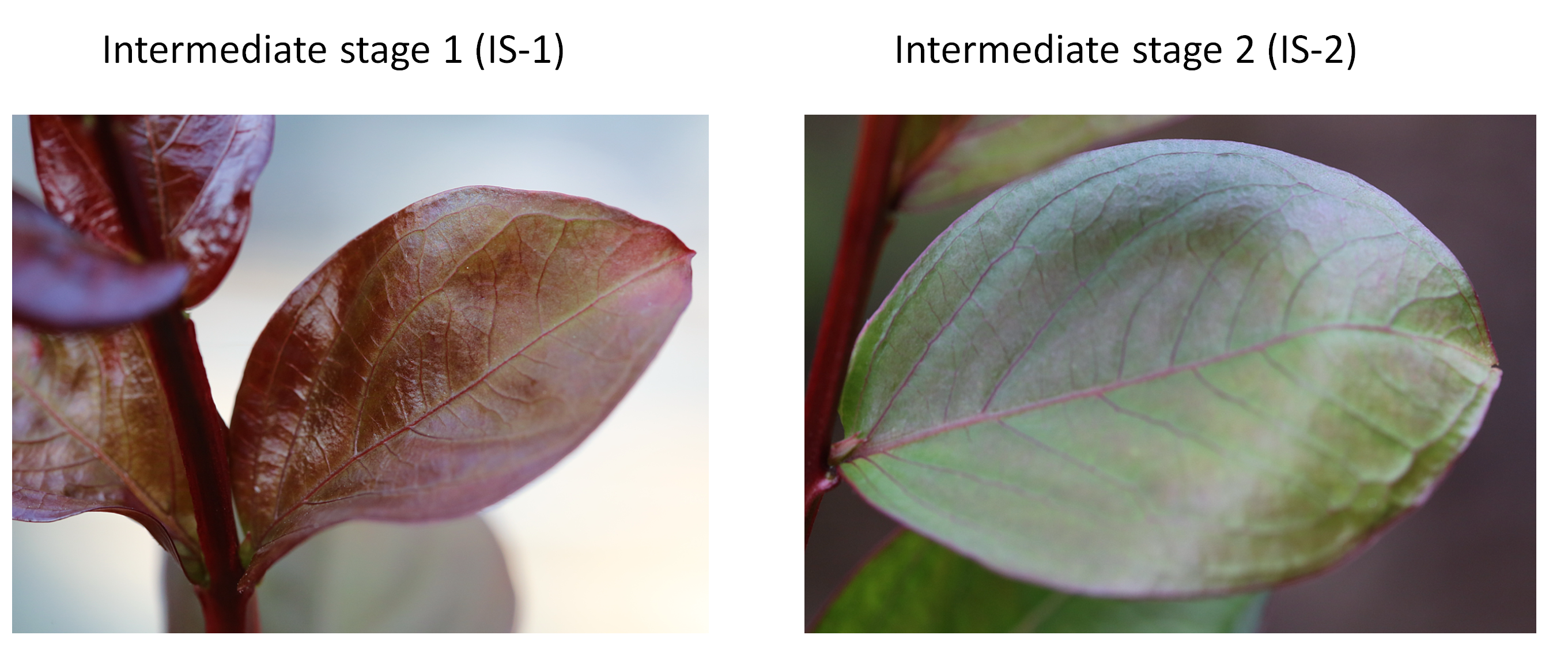

Supplement: Supplementary file 1 [file ijms-20-05636-s001.zip › Figure S2.tif]

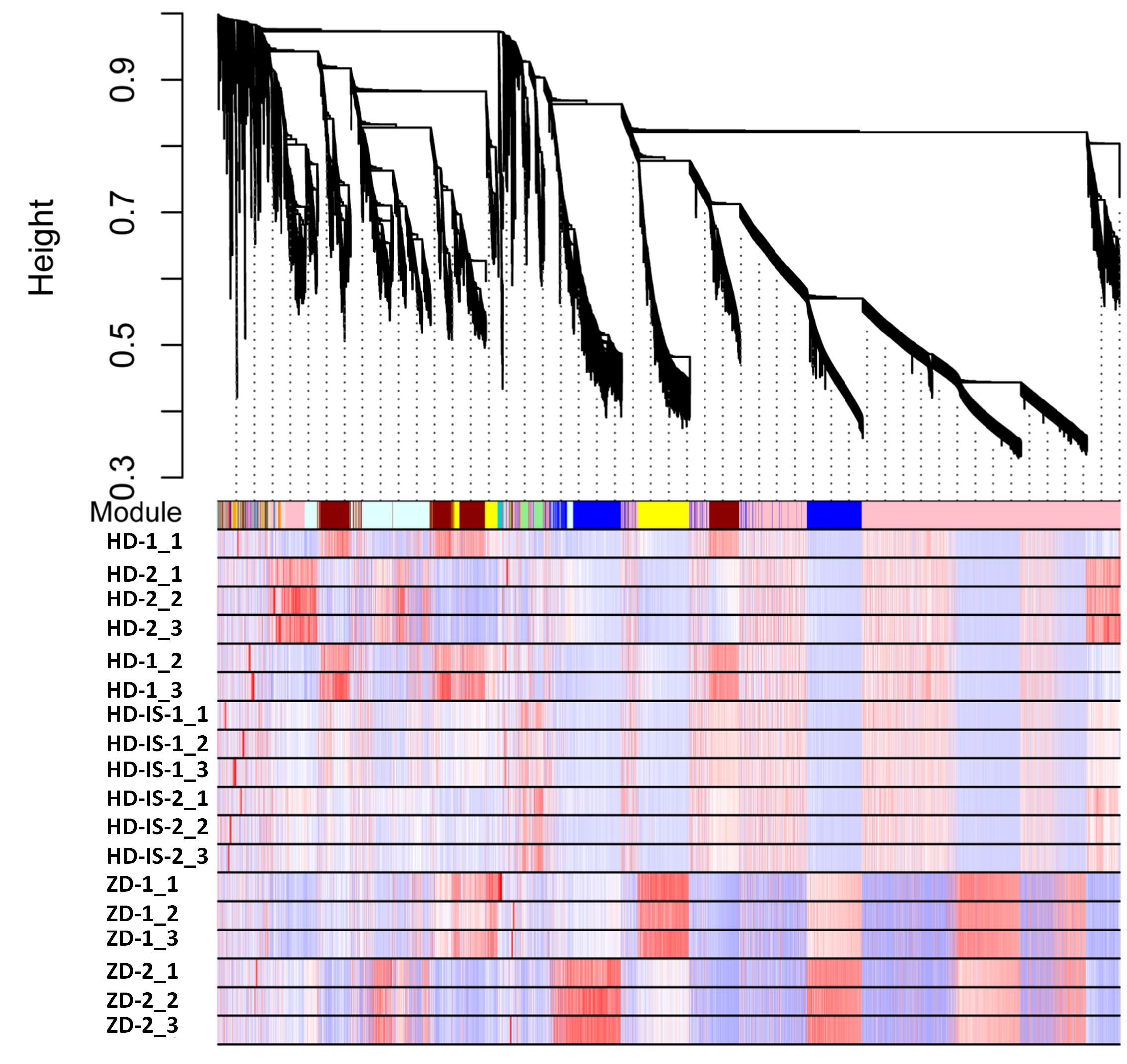

Supplement: Supplementary file 1 [file ijms-20-05636-s001.zip › Figure S3.tif]

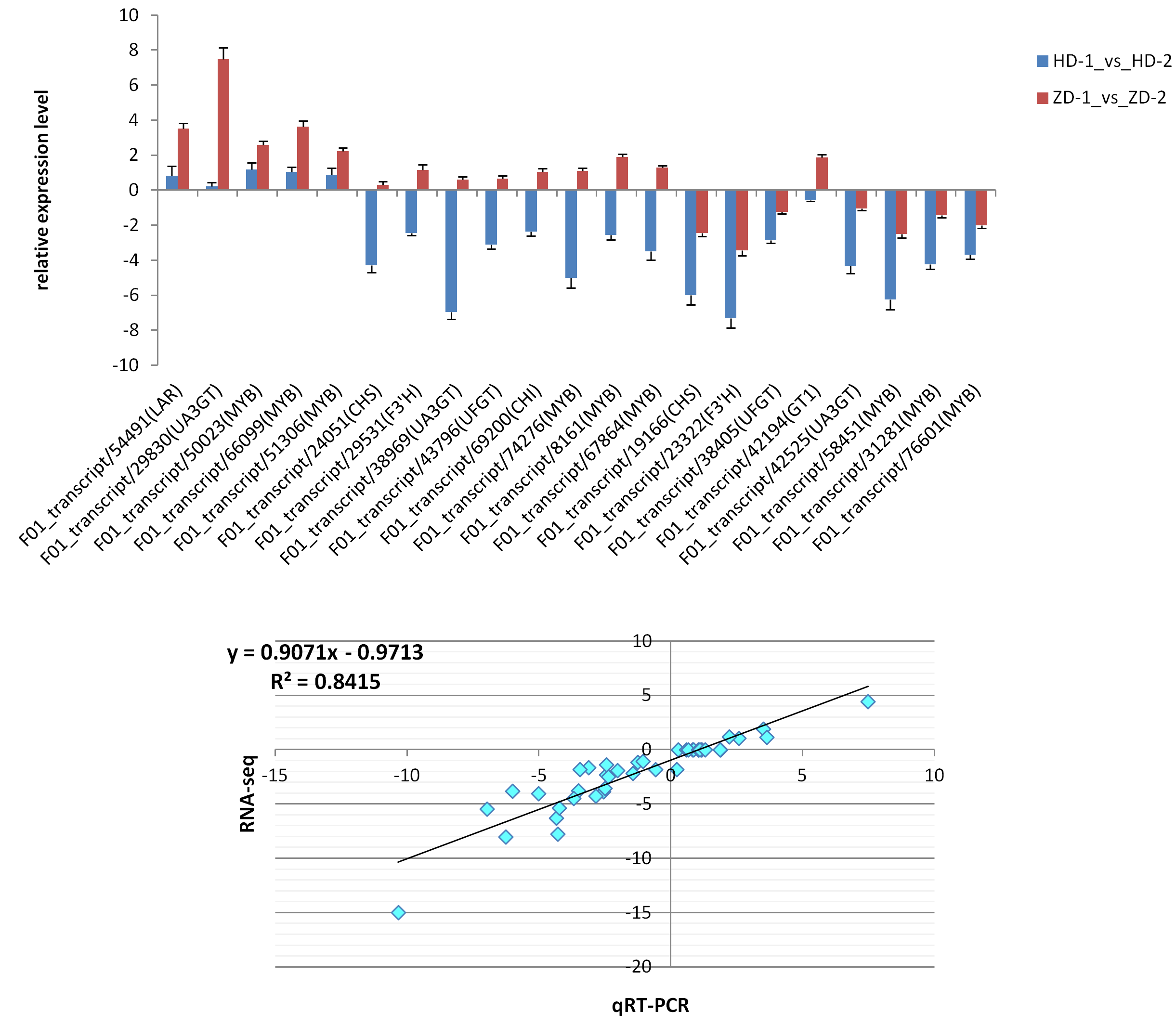

Supplement: Supplementary file 1 [file ijms-20-05636-s001.zip › Figure S4.tif]
